# The roles of long noncoding RNAs in breast cancer metastasis

**DOI:** 10.1038/s41419-020-02954-4

**Published:** 2020-09-14

**Authors:** Lingxia Liu, Yu Zhang, Jun Lu

**Affiliations:** 1 The Key Laboratory of Molecular Epigenetics of Ministry of Education (MOE), Northeast Normal University, Changchun, China; 2 The Institute of Genetics and Cytology, Northeast Normal University, Changchun, China

**Keywords:** Breast cancer, Cell migration

## Abstract

Breast cancer is the most significant threat to female health. Breast cancer metastasis is the major cause of mortality in breast cancer patients. To fully unravel the molecular mechanisms that underlie the breast cancer cell metastasis is critical for developing strategies to improve survival and prognosis in breast cancer patients. Recent studies have revealed that the long noncoding RNAs (lncRNAs) are involved in breast cancer metastasis through a variety of molecule mechanisms, though the precise functional details of these lncRNAs are yet to be clarified. In the present review, we focus on the functions of lncRNAs in breast cancer invasion and metastasis, with particular emphasis on the functional properties, the regulatory factors, the therapeutic promise, as well as the future challenges in studying these lncRNA.

## Facts

- LncRNAs can function as promoters or inhibitors of breast cancer metastasis.
- LncRNAs regulate metastasis at multiple levels, including transcription, translation, epigenetic modifications, and signaling pathway.
- LncRNA expression is regulated by both transcriptional and posttranscriptional factors during breast cancer metastasis.

## Open questions

- The same lncRNA may play dual roles during breast cancer metastasis. How do they perform the opposite function?
- In vivo identification of lncRNA biomarkers for organ-specific breast cancer metastases.
- The potential applications of lncRNAs as biomarkers or therapeutic targets.

## Background

**Fig. 1:**
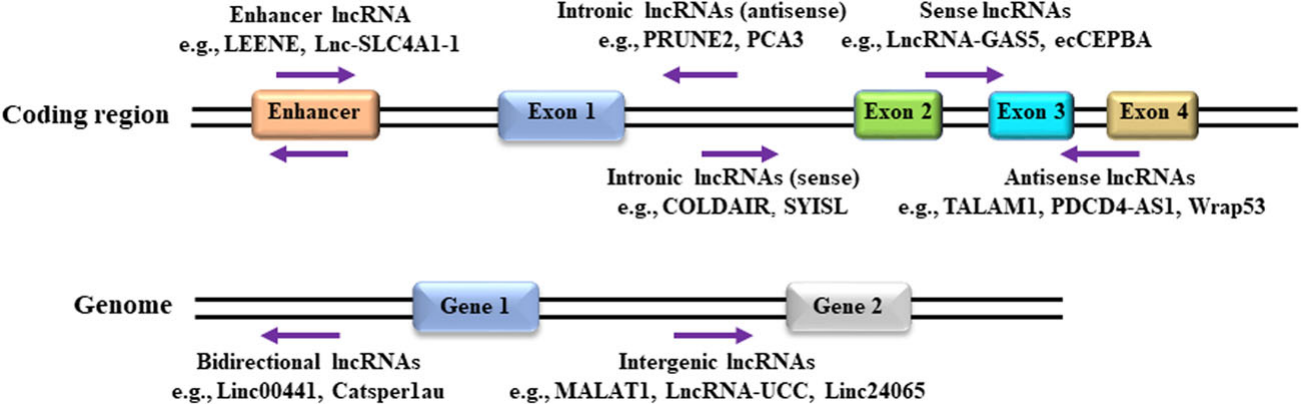
The classification of lncRNAs based on their locations of transcripts from genome. LncRNAs are divided into six groups, i.e., sense, antisense, intronic, bidirectional, intergenic, and enhancer RNAs.

Breast cancer is the most commonly diagnosed cancer in women worldwide^1,2^, and the distance metastasis is the leading cause of poor survival^3,4^. The molecular events that drive tumor cells to gain the metastatic characteristics have been extensively studied, resulting in significant improvements in diagnostic and prognostic approaches. However, a high incidence of death caused by breast cancer is still a paramount challenge. Therefore, elucidation of the novel metastasis-related molecular mechanisms is of great importance to improve the outcome of breast cancer treatments. Recently, many studies have revealed the close association of long noncoding RNAs (lncRNAs) with breast cancer metastasis^5–9^. LncRNAs belong to a large class of noncoding RNAs with the length >200 nucleotides, and they are involved in numerous biological processes, including cancer cell invasion and metastasis^10,11^. LncRNAs can be divided into six categories based on their gene loci, characteristics, and relationship with their neighbor genes^10,12^ as follows (Fig. 1). (1) Intergenic lncRNAs originate from DNA sequences between two protein-coding genes, such as MALAT1^13^, Linc-ROR^14^, and ANCR^15^; (2) intronic lncRNAs originate from introns of protein-coding genes, such as PRUNE2^16^, PCA3^17^, COLDAIR^18^, and SYISL^19^; (3) sense lncRNAs overlap with one or more introns and exons of different protein-coding genes, such as LncRNA-GAS5^13^ and ecCEPBA^20^; (4) antisense lncRNAs originate from an opposite direction to a protein-coding gene, such as TALAM1^21^, PDCD4-AS1^22^, and Wrap53^23^; (5) enhancer lncRNAs originate from the regions of promoter enhancer, such as LncRNA-LEENE^24^ and Lnc-SLC4A1-1^25^; and (6) bidirectional lncRNAs exist in proximity of a coding transcript of the opposite strand, such as Linc00441^26^ and Catsper1au^27^. So far, lncRNAs have been shown to exert auxiliary functions either to tumor suppression or to tumorigenesis. Specifically, in breast cancer, increasing evidence has strengthened the notion that lncRNAs play an important role in regulating breast cancer metastasis^13,28,29^. This article aims to outline the implications of lncRNA in the landscape of breast cancer metastasis according to their influence in the gain or loss of metastatic signatures.

## The dual function of lncRNAs in breast cancer metastasis

Several studies indicated that lncRNAs play essential roles in different stages of cancer development and progression. Aberrant expression of lncRNAs is correlated with breast cancer metastasis. Practically, lncRNAs can function as promoters or inhibitors of breast cancer cell invasion and metastasis.

### LncRNAs function as promoters of breast cancer cell invasion and metastasis

Some lncRNAs are abnormally upregulated in a variety of breast cancer cells^30–33^. Many studies have indicated that several lncRNAs, such as HOTAIR, linc-ROR, and BCAR4, are upregulated and they promote breast cancer invasion and metastasis (Table 1). HOTAIR is the first lncRNA found to be able to promote tumor progression. An early study showed that HOTAIR was highly expressed in metastatic breast cancer tissues and promoted breast cancer lung metastasis^34^. Mechanistically, HOTAIR recruited PRC2 complex to specific target genes genome wide, resulting in altered histone H3 lysine 27 methylation, an epigenetic silencing of metastasis suppressor genes, which increased cancer invasiveness and metastasis^34^. Thus, the interdependence between HOTAIR and PRC2 has therapeutic implications for breast cancer metastasis. However, the precise molecular mechanisms by which HOTAIR regulates PRC2 are unclear. Moreover, HOTAIR upregulation was strongly correlated with lymph node metastasis in triple-negative breast cancers (TNBCs)^35^, though the details of how it regulates breast cancer lung metastasis and lymph node metastasis are not yet clear. Apparently, further research is demanded to elucidate the roles and precise actions of HOTAIR in the occurrence, development, and progression of breast cancer metastasis. Remarkably, our previous study identified, for the first time, a novel role of linc-ROR in control of epithelial–mesenchymal transitions (EMT) and metastasis in breast cancer cells^36^. We showed that overexpression of linc-ROR induced EMT, and promoted migration and invasion in breast cancer cells, while knockdown of linc-ROR in breast cancer cells repressed breast cancer lung metastasis in vivo in immunodeficiency mice. Also, our data indicated that linc-ROR functioned as a ceRNA to regulate mir-205 activity to prevent the mir-205 target genes from degradation, leading to breast cancer lung metastasis. Meanwhile, Jiayan et al.^37^ discovered that linc-ROR also acted as a decoy lincRNA to block the recruitment of chromatin regulatory factors (G9A methyltransferase), abolished histone H3K9 modification of the TESC (Tescalcin) promoter, resulting in abnormal breast cancer metastasis. However, further studies are required to validate the genome-wide profile of linc-ROR occupancy for exploring more linc-ROR-targeting epigenetic marks in breast cancer metastasis. Moreover, linc-ROR is also a strong negative regulator of p53 through direct interaction with the heterogeneous nuclear ribonucleoprotein I (hnRNP I), leading to the inhibition of p53-mediated cell cycle arrest and apoptosis^38^. Based on this, the RoR–hnRNP I–p53 axis may also play a vital role in breast cancer metastasis. LncRNA BCAR4 is highly expressed in advanced breast cancer patients and contributes to breast cancer metastasis mediated by chemokine-induced binding of BCAR4 to two transcription factors (SNIP1 and PNUTS) with extended regulatory consequences, licensing the activation of a noncanonical hedgehog/GLI2 transcriptional program that promotes cell migration^39^. Based on the fact that hedgehog signaling pathways are aberrantly activated in a variety of cancer types, we speculate that lncRNA BCAR4 may also play an important role in hedgehog signaling pathways to regulate other cancer metastasis. Accumulating evidence has validated that a large number of lncRNAs are able to promote breast cancer metastasis, these include lnc-SLC4A1-1^40^, TINCR (terminal differentiation-induced noncoding RNA)^41^, Lnc-BM^42^, BLACAT1^43^, H19^44,45^, and many others^13,46,47^. These findings clearly indicate that specific lncRNAs are essential players in enhancing the ability of the breast cancer cell invasion and metastasis.

**Table 1:** LncRNAs promote migration, invasion, and metastasis of breast cancer.

LncRNA	Functions	Functional mechanism	Refs.
HOTAIR	↑ Migration, invasion, metastasis	Scaffold molecules, histone modification, guide, chromatin remodeling, STAT3 pathway, p53 pathway	^85,90,97,152^
BCAR4	↑ Migration, invasion, metastasis	Histone modification	^39^
Linc-RoR	↑Migration, invasion, metastasis	ceRNA, transcriptional regulation, decoys, histone modification, protein translation	^36,37,153^
Lnc-SLC4A1-1	↑ Migration, invasion	NF-κB pathway Enhancer lncRNA	^40^
TINCR	↑ Migration, invasion, metastasis	ceRNA	^41^
Lnc-BM	↑ Migration, invasion, metastasis	STAT3 pathway	^42^
NEAT1	↑ Migration, invasion	ceRNA	^154^
BORG	↑ Invasion, metastasis	Transcriptional regulation	^155^
LincIN	↑ Migration, invasion, metastasis	Transcriptional regulation	^80^
SUMO1P3	↑ Migration, invasion	cRNA	^55,156^
Lnc015192	↑ Migration, invasion, metastasis	ceRNA	^157^
Lnc01638	↑ Migration, invasion, metastasis	Protein stability	^158^
ARNILA	↑ Migration, invasion, metastasis	ceRNA	^159^
lncRNA HIT	↑ Migration, invasion, metastasis	TGFβ pathway	^99^
LncRNA-ATB	↑ Migration, invasion, metastasis	TGFβ pathway, ceRNA, mRNA stability	^160^
CCAT2	↑ Migration, invasion, metastasis	TGFβ pathway	^161^
TUG1	↑ Migration, invasion, metastasis	Caspase signaling pathway	^98^
MALAT1	↑ Migration, invasion, metastasis	ceRNA, alternative splicing	^76,162^
HOST2	↑ Migration, invasion	ceRNA	^163^
LncRNA RP1	↑ Migration, invasion	Protein translation	^106^
LINP1	↑ Migration, invasion, metastasis	p53 pathway	^164^
MAYA	↑ Migration, invasion, metastasis	Hippo pathway	^91,92^
LncRNA H19	↑ Migration, invasion, metastasis	PI3K/AKT pathway TGFβ pathway ceRNA	^45^
BLACAT1	↑ Migration, invasion, metastasis	ceRNA	^43^
LncRNA 91H	↑ Migration, invasion, metastasis	Histone modification DNA methylation	^13,86^
UCA1	↑ Migration, invasion, metastasis	Wnt pathway	^94^
BDNF-AS	↑ Metastasis	Scaffold molecules Protein stability	^165^

### LncRNAs function as inhibitors of breast cancer cell invasion and metastasis

Meanwhile, studies revealed that in contrast to those metastasis-promoting lncRNAs, other lncRNAs, such as MALAT1, NKILA, and ANCR can suppress breast cancer metastasis (Table 2). LncRNA MALAT1 is one of the most conserved lncRNAs; and it is abundant in normal tissues^48–50^. MALAT1 was first recognized as a biomarker to predict metastasis and survival in early-stage non-small cell lung cancer^51^. In breast cancer, knockout of MALAT1 induced the metastatic ability. Conversely, MALAT1 overexpression inhibited breast cancer metastasis in transgenic, xenograft, and syngeneic models^52^. However, some knockdown studies using antisense oligonucleotide (ASO) of MALAT1 in breast cancer cell lines and animal models demonstrated impaired cell migration, and significantly reduced metastasis^53^. These seemingly contradictory findings may implicate the complexity and intricacies of the roles of MALAT1 in breast cancer metastasis. Besides, lncRNA NKILA was shown to suppress breast cancer metastasis and was associated with poor patient prognosis^54^. Mechanistically, NKILA binds to NF-κB/IκB complex and inhibits NF-κB signaling through masking the phosphorylation sites of IκB and stabilizing the complex, leading to breast cancer metastasis. In addition, NKILA differs from those scaffold lncRNAs by directly regulating signal transduction without mobilizing other regulatory proteins, further illustrating the novel mechanisms of how these lncRNAs interact with the signaling proteins. Our previous study has also demonstrated that lncRNA ANCR was a repressive regulator of breast cancer metastasis in vivo in immunodeficiency mice^29,55^. We also found that ANCR interacted with EZH2 and facilitated CDK1 binding with EZH2 to promote its phosphorylation, leading to EZH2 degradation^29^. Nevertheless, the details of how ANCR physically interacts with EZH2 and how this interaction promotes CDK1 binding with EZH2 still remain unclear, and needs further investigations. In addition, our study unraveled another mechanism of ANCR action, i.e., ANCR was involved in modulating TGF-β pathway to suppress breast cancer lung metastasis^55^. Significantly, we and others have confirmed that the mRNA and protein levels of EZH2 remained unchanged, when treated with TGF-β1^55,56^. This fact suggests that ANCR may participate in the TGF-β1-induced metastasis through other mechanisms than EZH2 degradation. Along with more intensive studies, an increasing number of tumor suppressor lncRNAs have been discovered, e.g., PDCD4-AS1^22^, LINC01133^57^, NORAD^58^, MEG3^59^, XIST^60–63^, etc. Growing evidence indicates that lncRNAs may become a promising biomarker in breast cancer metastasis.

**Table 2:** LncRNAs inhibit migration, invasion, and metastasis of breast cancer.

LncRNA	Functions	Functional mechanism	Refs.
MALAT1	↓ Migration, invasion, metastasis	Scaffold molecules, transcriptional regulation, PI3K/AKT pathway	^52,95^
NKILA	↓ Migration, invasion, metastasis	Protein stability, transcriptional regulation, scaffold molecules, NF-κB pathway	^54^
ANCR	↓ Migration, invasion, metastasis	Protein stability, transcriptional regulation, TGFβ pathway	^29^
PDCD4-AS1	↓ Migration	mRNA stability	^22^
LINC01133	↓ Migration, invasion, metastasis	Transcriptional regulation	^57^
XIST	↓ Migration, invasion, metastasis	ceRNA	^61^
CASC2	↓ Migration, invasion, metastasis	ceRNA	^166^
MEG3	↓ Migration, invasion	PI3K/AKT pathway	^127^
NORAD	↓ Migration, invasion, metastasis	YAP pathway Decoy	^58^
GAS5	↓ Invasion	ceRNA	^96^
LIMT	↓ Migration, invasion, metastasis	EGF pathway	^93^

## The functional mechanisms of lncRNAs in breast cancer metastasis

Understanding how lncRNAs mediate cancer invasion and metastasis has been the aim of many researches over the time of nearly a decade. Interestingly, the available data suggest that lncRNAs seem to regulate metastatic gene regulation and function at various levels from transcriptional to translational^64^. Furthermore, lncRNAs are also involved in a variety of cell signaling pathways to modulate breast cancer metastasis. The following is a summary of the current understanding of the functional mechanisms of lncRNAs linked to breast cancer cell invasion and metastasis.

### Transcriptional regulation

**Fig. 2:**
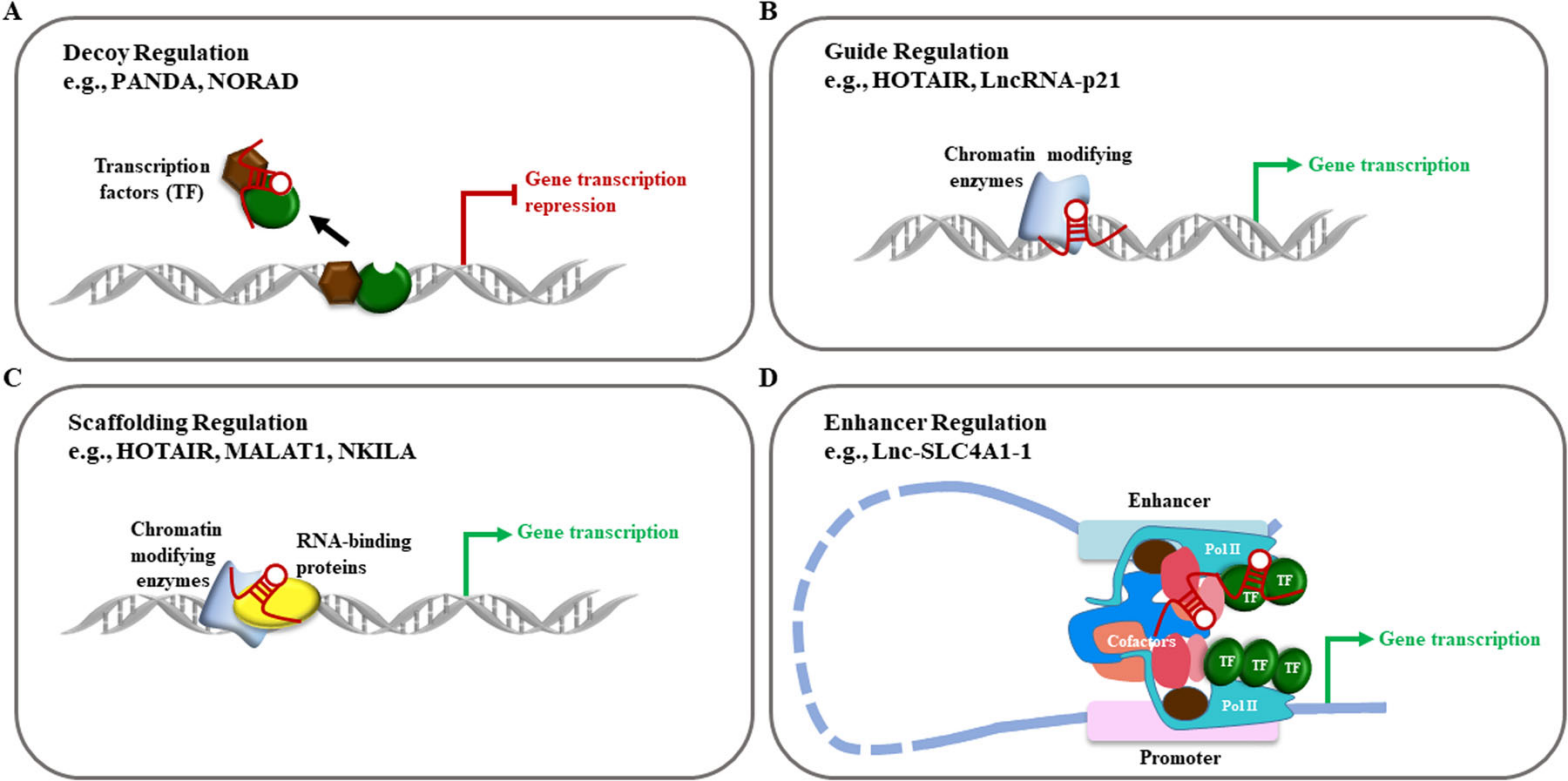
Functional mechanisms of lncRNAs at transcriptional levels. **a** LncRNAs act as decoys titrating away transcription factors and other proteins away from chromatin. **b** LncRNAs act as guides recruiting chromatin-modifying enzymes to target genes. **c** LncRNAs act as scaffolds for RNA-binding proteins to recruit chromatin-modifying complexes. In all cases, the binding of the enzymes or the recruiting factors are shown. **d** LncRNAs act as enhancer RNAs stabilizing looping and recruitment of transcriptional regulators, cofactors, and RNA Pol II, further increasing transcription of the associated gene.

At the transcriptional level, lncRNAs can act as decoys, guides, scaffold molecules, and enhancers to promote or inhibit gene expressions in breast cancer metastasis (Fig. 2). For instance, lncRNAs Lethe^12,65^, NORAD^58^, and PANDA^66^, act as “decoys” that bind miRNAs or proteins in the nuclei to mimic and compete with their consensus DNA-binding motifs, or act as decoy microRNA-binding sites like ceRNAs to control the functions of regulatory miRNAs^67^ (Fig. 2a). In addition, the “guiding lncRNAs”, like KCNQ1OT1^68^, HOTAIR^12^, and lincRNA-p21^69^, interact with transcriptional co-regulators or chromatin regulatory protein complexes, and recruit them to a specific DNA region to modulate transcription^70^ (Fig. 2b). Furthermore, lncRNAs can also act as scaffold molecules to form ribonucleoprotein complexes and thereby cooperatively control gene regulation^71^, such as MALAT1^72^, HOTAIR^73^, and LINP1^74^ (Fig. 2c). Interestingly, the “enhancer RNAs”, such as Lnc-SLC4A1-1^40^ and lncRNA-EBIC^75^, are transcribed from enhancer regions, interact with the enhancer–promoter, further regulating the transcription of the protein-coding genes (Fig. 2d).

### Posttranscriptional regulation

**Fig. 3:**
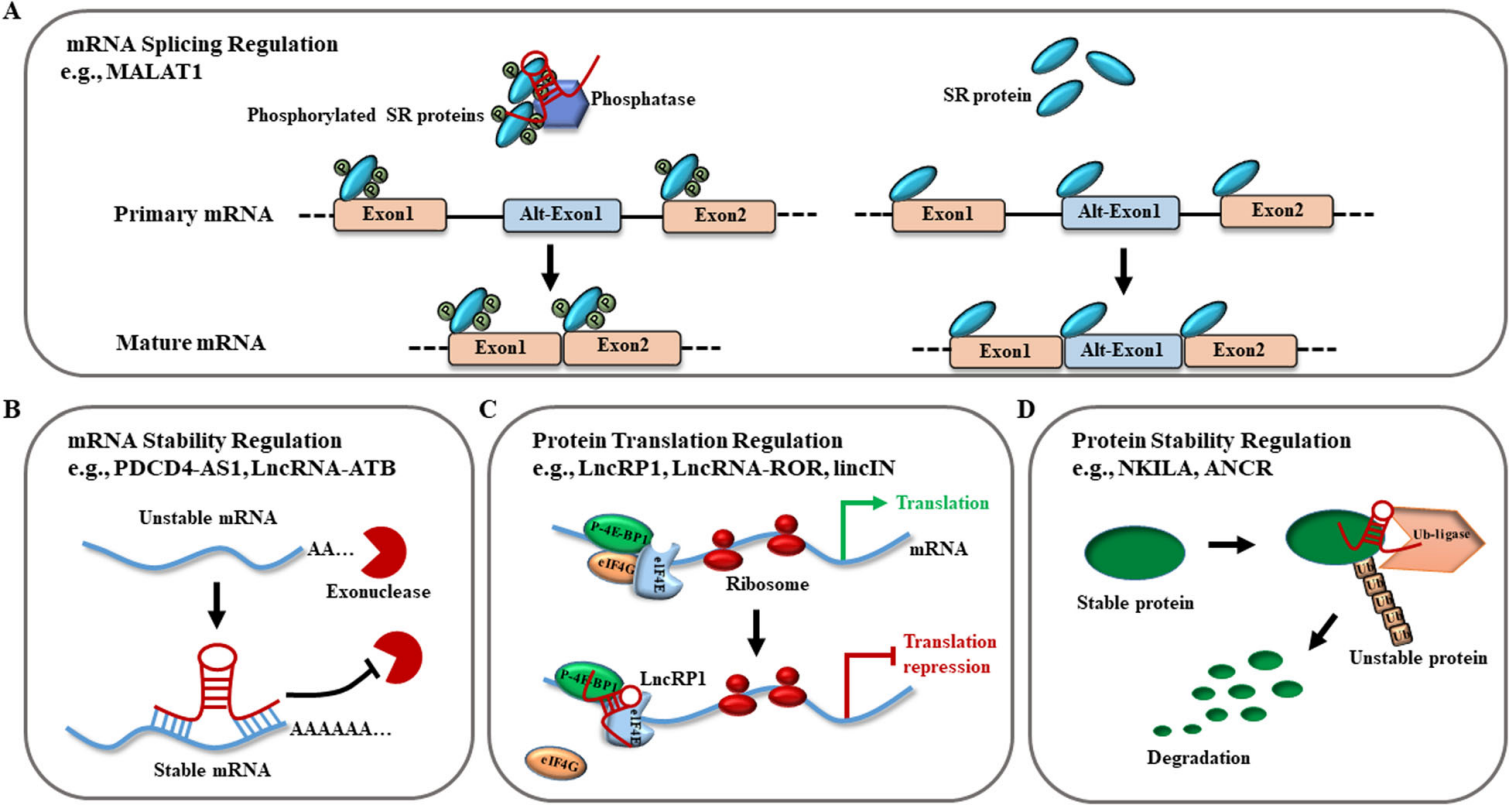
Functional mechanisms of lncRNAs at posttranscriptional levels. **a** LncRNAs change the splicing pattern of the pre-mRNA by regulating phosphorylation forms of the serine/arginine splicing factors. **b** LncRNAs stabilize mRNAs by forming RNA duplex, thereby preventing degradation. **c** LncRNAs regulate protein translation through recruiting the translation-related protein complex. **d** LncRNAs promote the degradation of proteins through recruiting the ubiquitin ligase.

At the posttranscriptional level, lncRNAs regulate mRNA splicing, mRNA stability, protein translation, and protein stability. One of such posttranscriptional mechanisms involves the RNA splicing regulated by lncRNAs (Fig. 3a). For example, the lncRNA MALAT1 expression was associated with tumor metastasis in various tumor types, including breast tumor^5^. Specially, MALAT1 interacted with the serine/arginine (SR) splicing factors to modulate SR distribution to nuclear speckles, further altering the pattern of alternative splicing for a set of pre-mRNAs^76^. Meanwhile, Bernard et al. reported that knockdown of MALAT1 modulated the recruitment of SR family pre-mRNA-splicing factors to affect the synapse formation^77^. An important question arising from these observations has been that how the SR protein-regulated alternative splicing is achieved in vivo in the context of specific cell or tissue types, or in response to extracellular signals. In addition, as antisense RNAs, lncRNAs can also affect the mRNA stability, which has been shown to be vital in breast cancer metastasis (Fig. 3b). For example, Wrap53, a natural antisense transcript of the tumor suppressor gene p53, increased endogenous p53 mRNA levels by targeting the 5′ untranslated region of p53 mRNA^78^. Likewise, PDCD4-AS1 stabilized tumor suppressor PDCD4 RNA by forming RNA duplex, and controls the interaction between PDCD4 RNA and RNA decay promoting factors, thereby contributing to breast cancer progression and migration^22^. In general, these studies have underscored the importance of antisense RNAs in breast cancer metastasis via its role in regulating the expression of a tumor suppressor sense partner. Future studies will unravel mechanistic roles of hundreds of other breast cancer-deregulated antisense RNAs in breast cancer metastasis. Protein translation regulated by lncRNAs is an important mechanism (Fig. 3c). Our previous study discovered that lncRNA-ROR promoted breast cancer progression and metastasis^36^, and it has been shown that lncRNA-ROR suppressed p53 translation by direct interaction with hnRNP I^38,79^. Similarly, lincIN played a key role in breast cancer cell invasion and metastasis through interacting with NF90, and it regulated p21 expression at the translation level^80^, although the details of how lincIN mediates breast cancer metastasis through the NF90-p21 pathway are yet to be specified. In addition, many studies have revealed that lncRNAs are crucial regulators of the stability of proteins that they interact with (Fig. 3d). For instance, we have shown that ANCR overexpression facilitated EZH2 ubiquitination to decrease the stability of EZH2 protein, resulting in breast cancer metastasis^29^. Likely, lncRNA-LET associated with NF90 protein to promote its ubiquitination and subsequent degradation^81^. Also, the lincRNA-p21 bound with HIF-1α and VHL to disrupt the VHL–HIF-1α interaction, thus to attenuate the VHL-mediated HIF-1α ubiquitination and caused HIF-1α accumulation^82^. Thus, lncRNAs participate in posttranscriptional regulation through a variety means, including mediating mRNA stability, mRNA splicing, protein translation, and protein stability.

### Epigenetic regulation

**Fig. 4:**
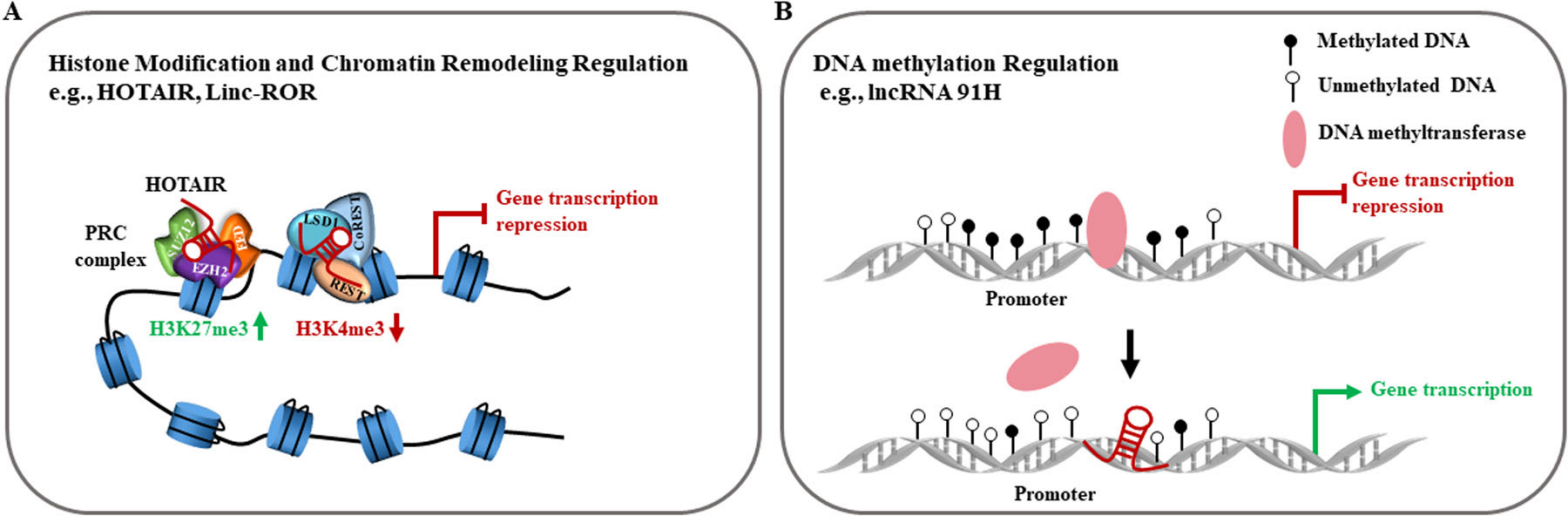
Functional mechanisms of lncRNAs at epigenetic levels. **a** LncRNAs regulate histone modifications or chromatin remodeling by interacting with PRCs or other chromatin-modifying proteins. **b** LncRNAs regulate DNA methylation in the promoter region of a downstream gene through inhibiting DNA methyltransferase recruitment.

Epigenetically, lncRNAs are involved in regulation of the modifications of histones (Fig. 4a), remodeling of chromatin structures (Fig. 4a), and DNA methylation (Fig. 4b). The important functions of lncRNAs are linked to epigenetic control of particular target genes, especially their repressive functions. Many lncRNAs, e.g., HOTAIR, linc-ROR, ANRIL, H19, and XIST, repress gene transcription by recruiting histone-modifying or chromatin-remodeling proteins^37,83,84^. In breast cancer metastasis, HOTAIR was associated with the PRC2 and LSD1 histone-modifying complexes to increase histone H3K27 methylation and H3K4 demethylation, resulting in suppression of target genes related with the anti-metastasis properties^85^. Also, Fan et al.^37^ have demonstrated that lncRNA-ROR occupied and activated the TESC gene promoter through repelling the histone G9A methyltransferase, and promoting the release of histone H3K9 methylation, leading to significant reduction of tumor growth and metastasis. In addition, lncRNAs also regulate DNA methylation to participate in tumorigenesis. For instance, LncRNA 91H at the H19/IGF2 locus is transcribed in H19 antisense orientation and named 91H. In breast cancer, 91H lncRNA prevents DNA methylation on the maternal allele at the H19/IGF2 locus, and thereby increases aggressive phenotype of breast cancer cells^13,86^. Furthermore, HOTAIR could induce miR-454-3p DNA methylation by recruiting EZH2 and DNA methyltransferase 1 to its promoter, thereby regulating gene transcription^87,88^. Thus, lncRNAs are involved in mediating epigenetic control of gene expression through the modifications of histones, remodeling of chromatin structures, and DNA methylation.

### Signaling pathway regulation

**Fig. 5:**
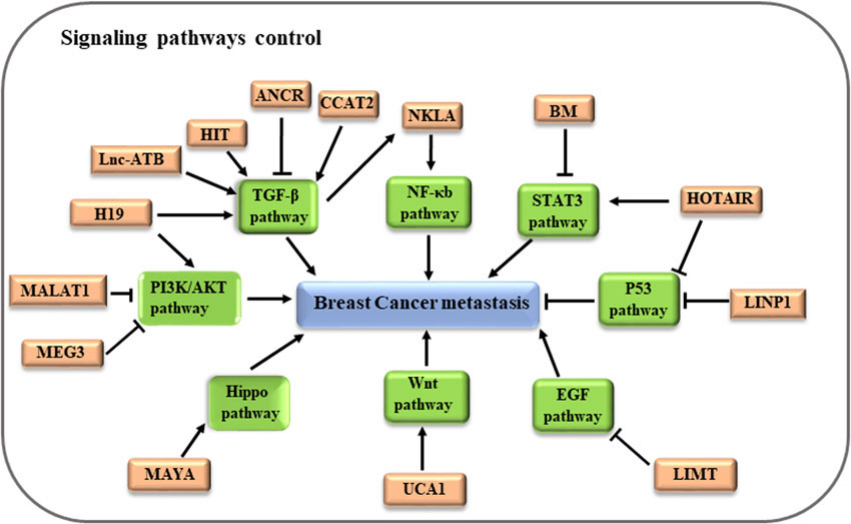
LncRNA-related signaling pathways in breast cancer metastasis. Aberrantly expression of lncRNA exerts an important impact on breast cancer metastasis by interacting with the TGF-β, STAT3, NF-κB, Hippo, EGF, Hippo, p53, and PI3K/AKT pathways.

Cellular signaling plays a key role in various cellular processes, including breast cancer metastasis, and several lncRNAs are known to be associated with some signaling pathways that are often critical to a complex cascade of breast cancer metastasis and metastasis-related gene expression. Emerging evidence suggests that the signaling lncRNAs can function as regulators of various pathways, such as TGF-β^55^, NF-κb^54^, STAT3^42,89,90^, Hippo^91,92^, EGF^93^, Wnt^94^, PI3K/AKT^95^, p53^96,97^, and others^58,98^, which play regulatory roles in breast cancer metastasis. In this review, we briefly discuss some pathways we believe important to breast cancer metastasis, including TGF-β, NF-κb, and STAT3 (Fig. 5).

#### TGF-β signaling pathway

Increasing data have shown that the TGF-β pathway plays a pivotal role in breast cancer metastasis and several lncRNAs have been defined in this process. For example, we previously found that lncRNA ANCR participated in TGF-β signal pathway through inhibiting RUNX2 expression, further inhibiting the breast cancer cell invasion and metastasis^55^. This work identified ANCR as a new TGF-β downstream molecule in breast cancer metastasis, and hence implicated that ANCR may become a prognostic biomarker and an anti-metastasis therapy target for breast cancer. However, the precise mechanism of how ANCR regulates RUNX2 expression in TGF-β pathway needs more intensive study. Moreover, Richards et al. demonstrated that lncRNA HIT regulated TGF-β pathway, i.e., lncRNA HIT downregulation suppressed TGFβ-induced breast cancer cell migration, invasion, and metastasis^99^. Collectively, these studies suggest that a subset of lncRNAs, such as lncRNA ANCR and HIT play a significant role in TGF-β pathway to regulate the breast cancer invasion and metastasis, and may be potential diagnostic and therapy biomarker in breast cancers.

#### NF-κB signaling pathway

NF-κB is a family of inducible transcription factors that are involved in inflammation, immunity, cell proliferation, and tumor metastasis through binding to the κB sequences^100,101^. Aberrant activation of NF-κB can influence invasion and metastasis in various cancers, including breast cancer^54^. Several lncRNAs play pivotal regulatory roles in the NF-κB pathway. LncRNA NKILA was first found upregulated by inflammatory cytokines TNF-α through NF-κB pathway in breast cancer. In details, NKILA could directly bind to NF-κB/IκB complex and inhibit NF-κB signaling to suppress breast cancer metastasis^54^. In another report, NKILA was shown to be upregulated by TGF-β to block NF-κB signaling, thereby suppressing the TGF-β-induced tumor metastasis in breast cancer^102^. Interestingly, the same lncRNA appears to be involved in different pathways; yet, the details of this phenomenon need further study.

#### STAT3 signaling pathway

STAT3 is first described as aggressive regulators in breast tumor biopsies^103^. Increasing evidence indicates that the STAT3 pathway plays an important role in breast cancer metastasis, and several lncRNAs (e.g., HOTAIR and Lnc-BM) participate in this process^42,89,90^. In breast cancer cells, Lnc-BM increased the STAT3-dependent expression of ICAM1 and CCL2, which regulated vascular co-option and recruitment of macrophages in the brain, respectively^42^. Recruited macrophages in turn produced oncostatin M and IL-6, and then activated the Lnc-BM/JAK2/STAT3 pathway to promote breast cancer brain metastases.

## The multiple-level regulation of lncRNAs in breast cancer metastasis

It has become increasingly clear that mammalian genomes encode numerous large noncoding RNAs. Regulation of the expression of conventional protein-coding genes takes place primarily at translation, posttranslational modifications, and protein stability. However, in breast cancer metastasis, the mechanisms that control the expression of lncRNAs are unclear. Present evidence suggests that similar to the expression of conventional protein-coding genes, lncRNAs expression is regulated by both transcriptional and posttranscriptional factors, excluding protein stability, in breast cancer metastasis.

### LncRNA expression is regulated by transcriptional factors

At transcriptional levels, multiple transcriptional factors are discovered to regulate the expression of lncRNAs, including c-MYC, KLF5, SP1, p53, NF-κb, Sox2, Oct4, Nanog, and ZNF143 during breast cancer metastasis^69,104–108^. c-MYC is a transcription factor that regulates a wide range of target genes that subsequently execute their multiple biological activities^109–111^. Importantly, in breast cancer metastasis, c-MYC is involved in the regulation of lncRNAs, e.g., lncRNA SNHG12, a direct transcriptional target of c-MYC^112^. As a result, the c-MYC-induced upregulation of lncRNA SNHG12 increases the ability of migration and metastasis in TNBC. This is considered as a new insight of c-MYC function, since previous studies on c-MYC-mediated transcriptional regulation have mainly focused on the coding transcripts. Specific links between c-MYC and lincRNAs have been investigated only recently^113–116^. Hart and his colleagues’ study revealed that a total of 534 lncRNAs were either upregulated or downregulated in response to c-MYC overexpression in human B cells, including SNHG15 and SNHG16^117^. During breast cancer metastasis, the regulatory effect of c-MYC on a broad segment of lncRNAs opens up a new area of c-MYC activity. The challenge is now to identify the breast cancer metastasis relevant lncRNA targets of c-MYC, and determine their functions. Furthermore, it has been reported that, during breast cancer metastasis, the transcription factor KLF5 recruits p300 and binds to lncRNA RP1 to enhance the activity of the RP1 promoter, and consequently enhances the RP1 expression^95^. The experiments also revealed that RP1 interacted with the complex p-4E-BP1/eIF4E to attenuate p27kip1 translation, and therefore promote breast cancer metastasis^95^. Importantly, both KLF5 and c-MYC could bind to the LINC00346 promoter, and coordinately enhance its expression to affect cell growth, migration, and invasion in gastric cancer^118^. This indicates that transcription factors may also coordinately regulate the expression of lncRNAs in breast cancer metastasis, and it needs further investigations. In addition, transcription factor Sp1 was implicated in an ample variety of essential biological processes, and has been proven important in cell growth, differentiation, apoptosis, and carcinogenesis^119^. Noticeably, lncRNA AGAP2-AS1 was upregulated and transcriptionally induced by SP1 in breast cancer metastasis^108^. Previous studies showed that SP1 can modulate many lncRNAs like LINC00673^120^, SPRY4-IT1^121^, LUCAT1^122^, and FTH1P3^123^ in a variety of malignancies in addition to breast cancer. Further studies will be required to address whether SP1 regulates other lncRNAs in addition to AGAP2-AS1 during breast cancer metastasis. Collectively, these observations demonstrate that the transcription factors play an important role in the regulation of lncRNAs transcription during breast cancer metastasis.

### LncRNAs expression is regulated by epigenetic mechanisms

Studies have demonstrated that some tumor-suppressive lncRNAs, e.g., LOC554202^124^ and MEG3 ^125,126^, are downregulated by high level of CpG methylation at their promoters during breast cancer metastasis. Specifically, the promoter-associated CpG islands of MiR-31 and its host gene lincRNA LOC554202 are heavily methylated in the TNBC cell lines, while they are hypomethylated in the luminal subtypes^124^. Loss of miR-31 and LOC554202 expression in TNBC cell lines is attributed to the hypermethylation of its promoter-associated CpG islands. Downregulation of miR-31 augments several steps critical in the invasion–metastasis cascade in breast cancer, indicating LOC554202 may also play an essential role in the invasion–metastasis cascade. Similarly, MEG3 expression is also under epigenetic control, i.e., aberrant CpG methylation associated with the expression of DNA methyltransferase DNMT3b^125,126^. Meanwhile, MEG3 overexpression inhibits breast cancer cell proliferation and invasion, suggesting that MEG3 expression may also be regulated by aberrant CpG methylation in breast cancer metastasis^127^. Expectedly, the roles of promoter methylation in the suppression of lncRNAs would become a focus of future study in breast cancer metastasis.

In addition to the DNA methylation, lncRNA expression is also controlled by histone modifications in breast cancer metastasis. TINCR is a spliced lncRNA, which produces a 3.7 kb transcript. A great deal of evidence shows that aberrant expression of TINCR is associated with a variety of human cancers, including breast cancer. During breast cancer cell metastasis, lncRNA TINCR upregulation is attributed to the transcriptional activation by the CREB-binding protein-mediated H3K27 acetylation enrichment^41^. Also, lnc-SLC4A1-1 is transcriptionally activated by H3K27 acetylation to stimulate the migration and invasion of breast cancer via activating CXCL8 and NF-κB pathway^40^. Thus, we speculated that other lncRNAs might be regulated by histone modifications in breast cancer metastasis.

### lncRNAs expression is regulated by posttranscriptional factors

Transcribed lncRNAs are further modulated through posttranscriptional processing, including alternative splicing, 5′capping, polyadenylation, and RNA editing, in breast cancer metastasis^128–131^. Through alternative processing of pre-mRNAs, a single mammalian gene often produces multiple mRNAs and protein isoforms, which may exert similar, different, or even opposite functions^132^. Over 90% of the multi-exon protein-coding genes undergo alternative splicing in humans^132^, and hnRNP E1 is widely documented for its role in the suppression of this process. Increasing evidence has identified that alternative splicing plays an important role in the regulation of lncRNAs expression in breast cancer metastasis. For example, PNUTS is a bifunctional RNA encoding both PNUTS-mRNA and lncRNA PNUTS, each eliciting distinct biological functions^131^. In breast cancer metastasis, the binding of hnRNP E1 to an alternative splicing site in the pre-RNA of PNUTS^131^ controls the generation of lncRNA PNUTS. Based on the existing data, we presume that certain antitumor agents whose pharmacological properties are linked to the blockage of transcription, might activate the alternative splicing of PNUTS to inhibit breast cancer metastasis. In addition, LncRNAs may also be mediated by 5′capping, polyadenylated, and RNA editing, similar to that of mRNA.

## LncRNAs as biomarkers and therapeutic targets

### LncRNAs used as breast cancer metastatic biomarkers

It is generally known that the distant metastasis is the major obstacle to the therapy of breast cancer. Therefore, identification of the metastasis-specific molecular biomarkers is essential for predicting metastasis. Several lncRNAs have been suggested to be dysregulated in breast cancer invasion and metastasis (Tables 1 and 2), indicating that lncRNAs have the potential to be biomarkers of diagnosis and prognosis in breast cancer. For example, the augmented expression of the lncRNA HOTAIR is associated with metastasis in breast cancer patients, suggesting its unique link with the patient prognosis^89^. Similarly, a clinical study revealed that high HOTAIR expression in primary breast tumors was significantly associated with worse prognosis, particularly in estrogen receptor (ER)-positive tumor samples. This finding suggested that HOTAIR might serve as an independent biomarker for the prediction of metastasis in ER-positive breast cancer patients^133^. To determine whether circulating HOTAIR can be used for breast cancer diagnosis, Lei and his colleagues detected HOTAIR in microliter amounts of serum, using a PCR-based direct detection assay^134^. Unexpectedly, they found that the DNA of HOTAIR in serum is a novel biomarker for breast cancer to distinguish breast cancer patients from healthy individuals. Although the aforementioned studies demonstrate that many lncRNAs, such as linc-ROR^36^ and ANCR^29^, are either upregulated or downregulated in breast tumor samples. One of the challenges with the clinical application of these lncRNAs is how to develop a convenient and quick technique to detect the target lncRNAs in breast cancer patients.

### Therapeutic promise of using lncRNAs targets

To date, many preclinical studies have confirmed that lncRNAs are essential contributors to tumor progression. For example, human-patient-derived xenograft model studies demonstrated that targeting lncRNA CamK-A robustly impaired breast cancer development; while clinically, the high expression of CamK-A indicated poor breast cancer patient survival rate, implicating its role as a therapeutic target^135^. Moreover, HOTAIR silencing significantly inhibited breast cancer cell metastasis in xenograft mouse models^136^. Also, a small-molecule compound AC1Q3QWB was found to act as a selective and efficient disruptor of HOTAIR-mediated recruitment of PRC2 in breast cancer patient-derived xenograft models^137^. In addition, lncRNA NKILA suppressed breast cancer metastasis in a xenograft mouse model; and low NKILA was associated with poor patient prognosis^54^. Along with more intensive studies, an increasing number of lncRNAs have been applied in preclinical studies, e.g., lnc-BM^42^, RP1^95^, linc-ROR^36^, etc. Thus, lncRNAs have now drawn intense research attention as prime targets for breast cancer therapy.

The development of RNA targeting therapeutics provides an opportunity to modulate lncRNAs for anti-breast cancer metastasis. The RNA-based strategies including RNA interference (RNAi), ASOs, clustered regularly interspaced short palindromic repeats (CRISPR)/Cas9, and CRISPR-Display to target lncRNAs, in the purpose of modulating the expression of lncRNAs. First, the lncRNA-specific siRNAs are used to inhibit the relevant lncRNA transcription^138^. For instance, downregulation of HOTAIR expression by using specific siRNA resulted in the reduction of tumor cell viability and invasiveness in breast tumors^134^. Although siRNA can efficiently inhibit cytoplasmic lncRNAs, these molecules have exhibited variable effects in targeting nuclear lncRNAs. Properly engineered ASOs is an effective tool that targets various RNAs regardless of the cellular location; and it applies single-stranded DNA or RNA molecules to direct the RNase H to target lncRNAs, further accelerating its degradation^139^. For instance, breast cancer progression can be inhibited by systemic knockdown of MALAT1 using ASO^53,140,141^. Thus, ASO may be more suitable for targeting the lncRNAs in the nucleus, whereas siRNA may be the better choice for targeting the lncRNAs in the cytoplasm. For many years, the technologies of lentivirus transfection have been widely used in the loss- and gain-of-function lncRNA experiments. Recently, a new approach, i.e., the CRISPR/Cas9, has emerged as a powerful tool to probe into the loss- and gain-of-function lncRNA studies. Furthermore, the RNAi technology is being supplemented by the CRISPR/Cas-9 system, which allows easier, more effective and multiple manipulation, from deletion of various parts of genomic lncRNAs loci, to insertion of promoters and exons^142^. This technology can be employed for targeted silencing of certain lncRNAs^9^. For instance, MALAT1 downregulation was achieved through use of CRISPR/Cas9 to study the MALAT1 effects on breast cancer metastatic incidence^52^. Moreover, the recent breakthrough of CRISPR/Cas9 system for genome editing in vivo^143–145^ holds great promise for targeting lncRNAs for cancer treatments. Recently, Shechner et al. developed a modified version of the CRISPR technique, termed the CRISPR-Display, which allows the insertion of RNA domains to DNA loci, thereby renders the identification of in *cis* behavior of lncRNAs^146^. This technology will hopefully provide a basis for the development of a wider array of approaches in lncRNA research.

## Prospect and challenges

In the past, lncRNAs are considered as “transcriptional noise”, i.e., nonfunctional RNAs^147^. However, an increasing number of studies have identified lncRNAs as indispensable contributors to many cellular activities including the breast cancer metastasis^148^. In this review, we have discussed several issues of lncRNAs, including their definition and classification, their dual functions for metastasis, their functional mechanisms, their regulatory factors, and the therapeutic promise in breast cancer metastasis. In comparison to the protein-coding mRNAs and miRNAs, our knowledge towards many details of lncRNAs is still limited.

First, the same lncRNAs have dual effects in breast cancer metastasis, and its functional basis is not clear. An example in this regard is the lncRNA MALAT1, which exerts opposite function in breast cancer metastasis, as revealed by Kim et al.^52^. They found that MALAT1 was a metastasis-suppressing lncRNA in NSG mice; however, Arun et al.^53^ and Li et al.^149^ reported that MALAT1 promoted the invasion and metastasis of breast cancer in the MMTV-PyMT mouse mammary carcinoma model and in 4T1 xenograft mice. Presumably, these seemingly contradictory phenomena could be explained as followed. The ultimate cause of the discrepancy may be due to the tumor microenvironment in vivo, although further work will be needed to identify the specific mechanism of the seemingly contradictory phenomenon. Second, lncRNAs are related with organ-specific breast cancer metastasis. Increasing evidence has shown that lncRNAs not only affect breast cancer lung metastasis, but also brain metastasis. Studies have shown that lncRNAs linc-ROR^36,148^, ANCR^29,55^, HOTAIR^34^, etc. regulate breast cancer lung metastasis. In addition, brain metastasis has been reported to be associated with several organ-specific lncRNAs, such as lnc-BM^42^, XIST^62^, NCT02915744 III, and NCT02000882 II^150^. These data implicate potential for further development of lncRNA biomarkers for organ-specific breast cancer metastases, and this warrants more attention of study. Third, lncRNAs may encode micropeptides to participate in breast cancer metastasis. Anderson et al. found a conserved micropeptide MLN encoded by a skeletal muscle-specific lncRNA, and that MLN functioned as an important regulator of skeletal muscle physiology^151^. This phenomenon also highlights the possibility that additional micropeptides may be encoded within the many RNAs currently annotated as noncoding, and suggests that distinction between mRNAs and lncRNAs may be less clear-cut than once thought. Meanwhile, few micropeptides have been reported to participate in breast cancer metastasis.

To conclude, the lncRNAs have opened a new window in studies of breast cancer; enhancing understanding of lncRNAs will certainly reinforce our knowledge of cancer development and management. In addition, the potential applications of lncRNAs as biomarkers and therapeutic targets have been documented, and this will become a hot topic in cancer diagnosis, prognosis, and therapeutics.
